# High-throughput sample processing for methylation analysis in an automated, enclosed environment

**DOI:** 10.1016/j.slast.2021.12.002

**Published:** 2021-12-17

**Authors:** Alejandro Stark, Thomas R. Pisanic, James G. Herman, Tza-Huei Wang

**Affiliations:** a Department of Mechanical Engineering. Johns Hopkins University. Baltimore, MD 21218, , USA; b Department of Biomedical Engineering. Johns Hopkins University. Baltimore, MD 21218, USA; c Johns Hopkins Institute for NanoBioTechnology. Baltimore, MD 21218, USA; d The UPMC Hillman Cancer Center, University of Pittsburgh, Pittsburgh, PA, 15213, USA

**Keywords:** Methylation-on-beads, Cytosine methylation, Epigenetics, Lab automation, Liquid handling platform applications

## Abstract

Variation in methylcytosine is perhaps the most well-studied epigenetic mechanism of gene regulation. Methods that have been developed and implemented for assessing DNA methylation require sample DNA to be extracted, purified and chemically-processed through bisulfite conversion before downstream analysis. While some automated solutions exist for each of these individual process steps, a fully integrated solution for accomplishing the entire process in a high-throughput manner has yet to be demonstrated. Thus, sample processing methods still require numerous manual steps that may reduce sample throughput and precision, while increasing the risk of contamination and human error. In this work, we present an integrated, automated solution for performing the entire sample preparation process, including DNA extraction, purification, bisulfite conversion and PCR plate preparation within in an enclosed environment. The method employs silica-coated magnetic particles that eliminate the need for a centrifuge or vacuum manifold, thereby reducing the complexity and cost of the required automation platform. Toward this end, we also compare commercial DNA extraction and bisulfite conversion kits to identify a protocol suitable for automation to significantly improve genomic and bisulfite-treated DNA yields over manufacturer protocols. Overall, this research demonstrated development of an automated protocol that offers the ability to generate high-quality, bisulfite-treated DNA samples in a high-throughput and clean environment with minimal user intervention and comparable yields to manual processing.

## Introduction

DNA methylation, namely the methylation of cytosine, is a fundamental mechanism of epigenetic regulation of gene expression [1,2]. Numerous studies have demonstrated that DNA methylation is involved in a wide range of cellular processes and plays particularly instrumental roles in embryonic development, cellular differentiation and the maintenance of cell phenotype [3,4]. On the other hand, abnormal changes in DNA methylation have been linked to many diseases [5], including multiple forms of cancer [6-10]. In fact, hundreds to thousands of cytosines become aberrantly hyper- or hypo-methylated during the typical development of most human cancers [11]. The close correlation between aberrant DNA methylation and the progression of disease, coupled with the inherent chemical and biological stability of DNA, has also led to significant interest in the use of methylation as a biomarker for cancer prediction, diagnostics and prognostics [12-15], particularly in challenging, noninvasively-collected samples such as serum [16-19], sputum [20,21], and urine [22].

Most methods for analyzing DNA methylation require a series of sample preparation steps starting with the extraction of genomic DNA (gDNA) from tissues, cells or cell-free genetic material derived from the biological specimen of interest. After purification, extracted DNA must then be processed by a method known as bisulfite conversion that chemically converts unmethylated cytosine residues to uracils, while leaving methylated cytosines unmodified. Thus, bisulfite conversion ultimately enables genomic analysis of epigenetic sequences by effectively translating DNA methylation patterns into changes in the primary sequence that can then be analyzed by standard molecular techniques such as polymerase chain reaction (PCR) [23] or sequencing [24].

The sample preparation steps (DNA isolation, bisulfite conversion and assay plate preparation) are typically performed sequentially using distinct protocols, often from different manufacturers, and often requiring sample transfer between steps. While automated protocols exist for some of these steps, they invariably require user intervention for some steps of the process such as pipetting, incubation and plate handling. The necessity of these manual steps introduces the possibility of human error as well as environmental contamination.

Alternative strategies based on microfluidic platforms have also been developed for streamlining DNA isolation [25,26] and bisulfite conversion [27,28] for specific sample volumes and types for low-throughput or point-of-care applications. However, these approaches are not well-suited to general use as they are not amenable for use with many sample types and volumes. Thus, an automated solution should ideally be able to accommodate a wide range of sample types of both large and small volume without compromising DNA yields or downstream performance.

Here, we present an automated solution for high-throughput benchtop sample processing including DNA isolation, bisulfite conversion, and assay preparation for immediate downstream analysis of DNA methylation. The automated solution requires only minimal user input for the initial setup and can perform all necessary steps within an enclosed environment. At the end of the process, the liquid handling platform (LHP) yields a qPCR-ready plate, which is then sealed by the user and transported to a qPCR thermal cycler for analysis.

## Materials and methods

### Instrument setup

**Figure 1.**
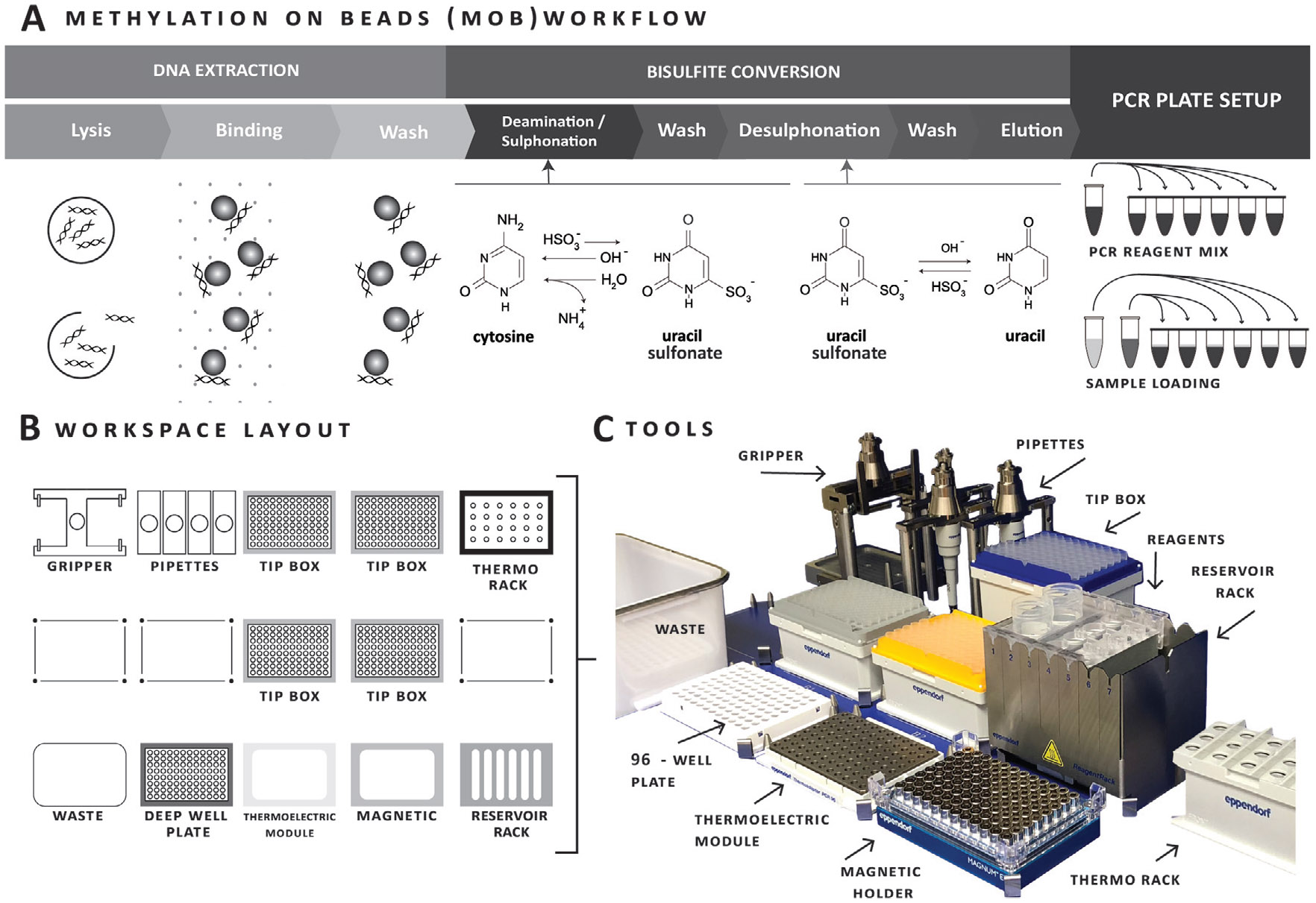
Automated extraction and processing DNA from biological specimens for methylation analysis. (A) The automated protocol represents an optimized and scaled up implementation of the methylation of beads (MOB) technique, described in prior publications. The robotic platform starts the MOB process by mixing the lysis buffer for cellular and protein digestion. Following gDNA binding to silica particles, the magnetic plate holder keeps the particles in the well while the supernatant is removed. The process is repeated with a wash buffer to further remove cellular debris. The process continues with bisulfite conversion, which starts with the addition of a low-pH bisulfite reagent for sulfonation and deamination. After washing, the process continues with the addition of a desulfonation buffer. A second wash step precedes elution of the gDNA. After elution, the gDNA solution is transferred to a clean tube. The robotic platform can prepare the PCR master mix from individual components and aliquot the mix and the samples into the PCR plate to prepare for qPCR analysis. (B) Layout of the EpMotion modules for the automated process. The necessary components (clockwise, starting at top left) for the MOB workflow on the EpMotion are the gripper for plate transport, the pipetting tools (total range from 0.2 μL to 1000 μL), disposable tips (blue) for the four different pipet sizes, a 1.5/2 mL rack (black) to hold the reagent tubes, a reservoir rack (grey) to hold larger 50 mL and 15 mL tubes, the magnetic adapter (purple) to pull down the beads within the working plate, a thermoelectric module (red) for heating and cooling the plate and the 1 mL deepwell plate (green) which is used as the main processing vessel for samples. (C) Image of the EpMotion setup highlighting the necessary components for automated MOB processing.

We designed an automated assay compatible with the Eppendorf (Hamburg, Germany) EpMotion 5075vtc liquid handling platform (LHP) equipped with a HEPA filtration system. The enclosed acrylic box contains all the tools within the work area and in conjunction with the filtration system, which provides an outward airflow, maintains external particles away from the samples, reagents and tools. We selected and placed all necessary tools and equipment (Figure 1) within the internal work area, including: the gripper tool for plate and accessory transport; for liquid handling, TS-10 (Eppendorf 5280000100), TS-50 (Eppendorf 96001010), TS-300 (Eppendorf 960001028) and TS-1000 (Eppendorf 96001036) single channel pipetting tools with their respective filtered tips (Eppendorf 0030014391- 10 μL, 0030014413 - 50 μL, 0030014456 - 300 μL, 0030014499 - 1000 μL). We equipped one of the two thermoelectric modules with a metal heat transfer adapter designed for the 96-Deepwell plate (Eppendorf 960002199) and the other with a 24-tube thermal rack (Eppendorf 960002080) for reagent storage. To enable magnetic particle separation, we placed a Magnum EX magnetic plate holder (Eppendorf 960066123) compatible with the 96-Deepwell plate. This magnetic plate holder contains an array of 96 magnetic rings that aggregate the magnetic particles on the well sidewall while leaving the bottom and central axis of the well clear of particles for liquid extraction.

**Figure 2.**
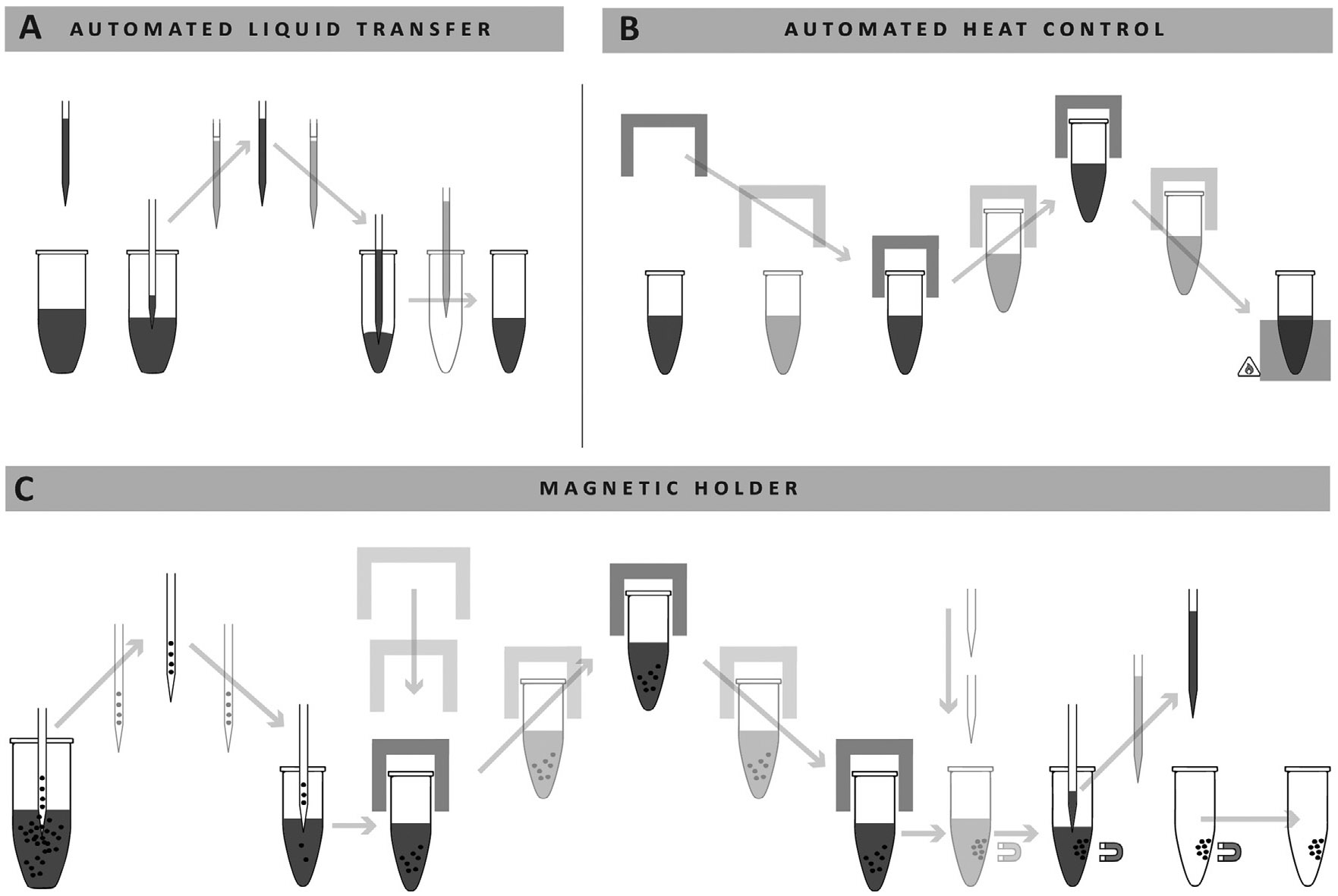
The automated platform enables processing by a combination of movements. Reagents and samples are transferred into and out of the vessels by the automated pipet system (A). The platform also utilizes a gripper to transport all reactions on a 96-well plate to the thermoelectric module (B) for incubation or onto a magnetic plate holder (C) for decantation of magnetic particles and removal of the supernatant without the need for manual intervention.

We prepared 200 ng of human genomic DNA (G1471, Promega Corporation, Madison, WI, USA) diluted in 150 μL total UltraPure PCR-grade water (10977015, Thermo Fisher Scientific, Waltham, MA, USA) as a control for all comparison experiments. Additionally, we validated the finalized automated process using human 150 μL sera (S7023-100ML, Sigma-Aldrich, St. Louis, MO, USA). We pipetted the samples into a 96-well, 1000 μL DNA LoBind Deepwell plate (951032808, Eppendorf) and placed it on the workspace. We arranged all necessary reagents in a variety of microcentrifuge and conical tubes including: 1.5 mL, 2.0 mL, 15 mL and 50 mL volumes and placed them on the appropriate thermal rack and reservoir rack modules as shown in Figure 2. We transferred all reagents containing DNA or RNA into DNA LoBind tubes (022431021, Eppendorf) before placing them in the machine and prepared, empty labeled DNA LoBind tubes for storage of processed gDNA and bisulfite-treated DNA (bstDNA) samples, and an empty 96-well plate (HSP-9601, Bio-Rad Laboratories, Hercules, CA, USA) for loading of PCR and bstDNA samples at the end of the process.

### Optimization of pipetting speed parameters

We determined the settings for liquid pipetting (aspiration, dispensing, mixing speed, and aspiration/dispensing during magnetic separation) experimentally to ensure the transported volume is precise. All pipetting tools were calibrated to manufacturer specifications, which are within ISO standard 8655-6 for normal operation [29,30], before testing. To maintain the accuracy and precision when transferring liquids with physical properties different than water, we determined the maximum speed empirically that would not disrupt the single liquid phase and affect the precision of the measured volume. With this consideration, we defined pipetting failure during aspiration as the presence of any droplets above the liquid level or bubbles within the liquid. For dispensing, we focused on ensuring the full measured volume was transferred to the destination well. Therefore, failure was defined as the presence of any liquid droplets remaining on the tip surface after dispensation. For mixing, we defined failure as the presence of any of these failure events or splashing within the destination well. We tested an additional case when pipetting liquid out of a well with magnetic particles fixed to the wall by the magnetic plate holder to ensure the stress from the liquid flow would not resuspend the particles and lead to their removal with the liquid phase. In this case, we defined failure as the speed at which either pipetting direction would disturb the particle pellet and cause dispersion. We increased the speed for each liquid by 5 mm/s increments until pipetting failure. The speed was then fine-tuned by reducing the speed at 1 mm/s intervals until the pipetting was successful again. For dispensing, we tested liquids with high adhesion (such as ethanol) using a blow-out option, which would push air beyond the measured volume to remove any leftover droplets. If the blow-out was successful in removing the droplets, we did not decrease the pipetting speed further and incorporated the blow-out into the programming. We repeated testing of the last successful parameter for 3 cycles to confirm reproducibility. If the pipetting failed in any repeat test, we reduced the speed by 1 mm/s until successful pipetting could be reproduced.

### Characterization of the automated process by qPCR

We assessed the precision and reproducibility of pipetting by comparing qPCR cycle of quantification (Cq) values and extrapolating the DNA yield according to a control DNA standard curve. We designed the primers for qPCR excluding cytosine regions to provide equal amplification of gDNA and bstDNA and allow for direct comparison of yield at both stages. To quantify them at a later stage, we designed the protocol to isolate a 10 μL gDNA aliquot before continuing bisulfite conversion and store the processed 50 μL bstDNA in separate tubes.

The machine transferred a 1 μL sample from each isolated tube to a 25 μL qPCR reaction containing a final buffer concentration of 16.6 mM ammonium sulfate (A418, Sigma-Aldrich), 67 mM TRIS (252859, Sigma-Aldrich), 6.7 mM magnesium chloride (M8266, Sigma-Aldrich), 10 mM *β*-mercaptoethanol (M6250, Sigma-Aldrich), 200 μM dNTPs (R0192, Thermo Fisher Scientific), 300 nM Primers, 100 nM TaqMan probe, and 1U/25 μL Platinum Taq polymerase (10966034, Thermo Fisher Scientific). We then loaded the PCR plate into a Bio-Rad CFX96 qPCR machine for amplification and analyzed the readout using the included CFX Manager software. We evaluated all statistical analysis on the resulting Cq values to identify the best performing kits and particles. To calculate the resulting gDNA and bstDNA yields from the Cq values obtained by the software we used a relative quantitation model. We calculated the lowest Cq value for the experiment set (Cqref) and calculated the relative quantity by assuming 100% PCR efficiency. By calculating 2^(Cqref-Cq) we found the normalized yields for each sample. We calculated the positive and negative error by calculating 2^(Cqref-Cq)-2^(Cqref-(Cq±SD)).

### Comparison of different extraction kits and silica particles

We compared a variety of DNA extraction kits: Liquid Lysis kit (NeoGeneStar, Somerset, NJ, USA), MagMax Cell-Free DNA Isolation Kit (A29319, Thermo Fisher Scientific), MagaZorb DNA Mini-Prep kit (MB1004, Promega Corporation), and Apostle MiniMax High Efficiency Isolation Kit (C40604, Beckman Coulter Inc., Sykesville, MD, USA). Each of these kits employs silica-coated magnetic particles allowing direct comparison to the previously-published MOB protocol[31], which uses reagents from the BioSprint15 DNA Blood kit (940014, Qiagen, Hilden, Germany). We programmed the EpMotion to follow the protocol provided by the manufacturer for each respective kit with the addition of a 200 μL mineral oil layer before the heat incubation step and an 800 μL 80% ethanol wash at the end to reduce potential PCR inhibitors. The final elution volume for all runs was set at 30 μL, from which 10 μL was used for quantification and the remaining 20 μL used for the downstream bisulfite conversion steps.

We performed bisulfite conversion on the extracted DNA using reagents from the Zymo Lightning Conversion Kit (D5030, Zymo Research, Irvine, CA, USA), according to the “Methylation on Beads” protocol as previously described[31,32] . Briefly, we mixed 20 μL of eluted gDNA from each sample with 130 μL of Lightning conversion reagent. We added a layer of 200 μL of mineral oil before incubation on the thermoelectric module at 98°C for 8 minutes followed by 60°C for 60 minutes. Following incubation, we removed the plate from the thermoelectric module and added 600 μL of M-Binding buffer to each well. The plate was incubated for 10 minutes at room temperature before being placed on the magnetic adapter and the aqueous phase was removed by magnetic decantation. The plate was then removed from the magnetic adapter and 500 μL of M-Wash buffer (D5001-4, Zymo Research) was added to each sample well, followed again by magnetic decantation. 300 μL of L-Desulphonation buffer (D5030-5, Zymo Research) was then added to each well and the plate was incubated at room temperature for 15 minutes and magnetically decanted. We washed the beads with 500 μL of M-Wash buffer followed by magnetic decantation and repeated this process a second time. After removal of this wash buffer, we removed the plate from the magnetic plate holder and added 800 μL of 80% ethanol. We placed the plate on the magnetic holder again and the ethanol removed. Following this, the plate was removed from the magnetic plate holder and 50 μL of elution buffer (D5001-6, Zymo Research) was added to each well. We incubated the plate at 70°C for 15 minutes before transferring back to the magnetic adapter. The remaining aqueous phase containing the eluted bstDNA was transferred into clean tubes for qPCR analysis.

We compared silica magnetic particles from each of the aforementioned kits, as well as Zymo MagBinding Beads and Absolute Mag Plain Silica Magnetic Particles 6 μm (SMP-UM15, Creative Diagnostics, Shirley, NY, USA), by running the Qiagen protocol using Qiagen reagents with the particles replaced with those from each of the other manufacturers at the corresponding recommended concentrations. We isolated aliquots from the gDNA extraction before bisulfite conversion and quantified by qPCR, as described above.

### Comparison with manual process

We selected the reagents from the Qiagen kit and the Zymo particles for the final protocol for automation. We performed the same protocol both manually and on the LHP with the same steps and volumes. For the automated assay, we placed the required samples and reagents into their appropriate positions in the machine before starting the run. This was the only manual step performed by the user after which the machine enclosure was shut, and the program run from the connected computer. The LHP then added 7.5 μL of pretreatment buffer, 6 μL of proteinase K and 5 μL of poly-A carrier RNA (Qiagen 1017647) to each well that contained 150 μL of serum. The LHP then mixed the samples by pipetting up and down for 5 cycles. A layer of 300 μL of mineral oil was added from the top and the plate was heated at 60°C for 30 minutes. Following this incubation, the LHP added 150 μL of Buffer AL and 150 μL of ethanol. The LHP then resuspended the silica-magnetic beads (D4100-2-12, Zymo Research), by pipetting up and down at 75 mm/s for 5 cycles and added 30 μL of beads to each sample. The LHP mixed each sample lysate by pipetting up and down for 5 cycles at 11 mm/s and incubated at room temperature for 10 minutes. The LHP then used the gripper to transfer the plate onto the magnetic adapter and the pipet tool to remove the aqueous phase from the bottom while the oil phase remained on top. The LHP removed the plate from the magnetic adapter and added 500 μL of buffer AW1 followed by mixing. The LHP then transferred the plate back onto the magnetic adapter and removed the aqueous phase. The LHP removed the plate from the magnetic adapter and added 500 μL of buffer AW2 followed by mixing. The LHP transferred the plate back to the magnetic adapter to remove the aqueous buffer from the bottom and repeat a cycle of adding and removing 500 μL of buffer AW2. This was followed by the addition of 500 μL of 80% ethanol, transfer to the magnetic adapter, and removal of the buffer. After removing the ethanol at the end of this cycle, the LHP added 40 μL of elution buffer to the beads. After incubating the sample at 70°C for 10 minutes, it transferred the plate back to the magnetic adapter. The LHP aliquoted a 5 μL sample of eluted DNA from this tube by pipetting from the bottom and placed in a clean tube. This sample was later used to measure gDNA extraction yield by PCR. The protocol for the bisulfite conversion portion was the same as described in the previous section. Lastly, the machine transferred the eluted bstDNA into clean tubes for storage at −80 °C.

For methylation specific PCR (MSP), we programmed the machine to prepare the PCR master mix by transferring the previously described volumes of 10X PCR buffer, dNTPs, primers, EvaGreen dye (31000, Biotium, Fremont, CA, USA), and polymerase from their respective tubes and mixing into a new tube. After preparing the mix, the machine aliquoted 24 μL of the PCR master mix and 1 μL of sample into each well in a 96-well PCR plate, followed by 5 cycles of up and down pipetting to thoroughly mix the reagents. We manually sealed the plates using an optical film (MSB1001, Bio-Rad) before running the PCR thermal cycling, consisting of a 98°C denaturation step for 30 s, a 60°C annealing step for 30 s and a 72°C extension step for 30 s, repeated for 40 cycles.

## Results and discussion

The primary aim of this work was to develop a fully automated and self-enclosed sample preparation method for DNA analysis comprised of DNA extraction, bisulfite conversion, and PCR plate preparation for DNA methylation analysis. To begin, we first sought to identify a liquid handling platform able to perform all steps of our previously-described Methylation-on-Beads (MOB) [31] sample processing technique for 96 samples in parallel and require minimal user intervention. We selected the EpMotion platform since it included all the necessary components for automating MOB, including pipetting, magnetic handling, heat incubation, and plate manipulation in a HEPA-filtered enclosure.

An overview of the automated MOB method is shown in Figure 1A. First, we facilitate lysis by adding buffer AL and proteinase K that work to release gDNA by disruption of cellular membranes as well as denaturation and digestion of any proteins contained in the sample. The lysis buffer also contains guanidine, a chaotropic salt, and is buffered at a low pH, both of which facilitate gDNA binding onto the silica coating of the magnetic particles. Cellular debris and other molecules remain in solution and are subsequently removed as we aspirate the supernatant. Meanwhile, the DNA-bound particles are immobilized by the magnetic plate holder inside the tube. We followed this binding step with two wash steps to further remove remaining debris, leaving the purified gDNA bound to the beads. The gDNA can optionally be eluted off at this point and quantified (by qPCR or other method) to obtain the sample gDNA yield.

For bisulfite conversion we selected a conversion kit with a fast bisulfite chemistry that would allow conversion to occur within an hour to avoid excessive evaporation without the use of adhesive lids or other methods not compatible with automation. While some kits have been reported to offer superior efficiencies, this was the best reported rapid kit[33]. For this part of the process, we mixed the purified gDNA sample with Zymo bisulfite conversion reagent and incubated at 98°C to denature the DNA and facilitate the chemical reaction. This reagent, in combination with a low pH, initiates conversion of cytosine into uracil sulfonate, while leaving methyl-cytosine intact. Following a buffer wash, a high pH, we added a desulfonation buffer to complete bisulfite conversion by hydrolyzing the uracil sulfonate into uracil. The DNA is bisulfite treated at this point and, after two wash steps, can be eluted into solution. The resulting purified bstDNA is now suitable for downstream methylation analysis techniques, such as bisulfite sequencing (BS-Seq)[34], quantitative methylation-specific PCR (qMSP)[35], methylation-sensitive high-resolution melt (MS-HRM)[36] and Discrimination of Rare EpiAlleles by Melt (DREAMing)[15].

The initial setup of the EpMotion instrument, including aliquoting of all reagents and samples into adequately sized tubes, is shown in Figures 1B and 1C. The liquid handling platform (LHP) employs optical detection of initial liquid volumes, which simplifies the setup process by automatically measuring reagent volumes. The user needs only to ensure provision of sufficient volumes for all reactions in the experiment, without the need to measure each one individually. Likewise, reagents can also be loaded in excess for multiple runs without the need to refill after each process. Once the run starts, the software keeps track of volumes without the need for additional optical measurement. One important optimization we performed during the programming process was to adjust the pipetting speed individually for each reagent. We found some of the default parameters were not appropriately calibrated. For example, pipetting of the lysis buffer with default settings introduced air into the reagent and caused a significantly lower volume of the reagent to be transferred. After determining the appropriate speed for each reagent (Supplementary Table 1), we programmed the complete routine and tested all the reagent transfers, confirming the accuracy of the pipetting process. We determined pipetting speed had to be significantly reduced during magnetic separation to avoid loss of beads when removing the supernatant. After an initial run, we further reduced the speed after noticing the presence of beads in the waste material from the accumulated supernatants. Another important consideration in the process is the use of 96-deepwell plates which allow for 1 mL volumes to be used in a reduced horizontal footprint compared to individual tubes. For larger volumes, a 24×10 mL deepwell plate that allows for processing of much larger sample volumes with the same footprint can be used with the appropriate magnetic plate holder and heat transfer adapter.

A notable disadvantage of the automated platform compared to a manual process is that corrections cannot be implemented mid-run and appropriate pipetting speeds for each step/reagent must be determined empirically. Similarly, we found that a major challenge in adapting manual assays to the automated platform is achieving reliable and repeatable pipetting rates without the visual feedback and adjustment that a manual process can provide. This was of particular importance when determining the pipetting speed for different liquids with different physical properties. We found some liquids particularly prone to bubbling, splashing, and adhesion to the pipet tips. To mitigate these effects, we reduced the pipetting speed and made use of a blow-out option in the programming where the pipet would aspirate air at the end of the pipetting process and then blow out any remaining liquid. The use of blow-out reduced the leftover liquid in fluids such as alcohol with a lower surface tension. So, while a faster processing speed is generally desirable, we ultimately prioritized pipetting consistency at a lower speed to maintain overall process reliability.

Having established the basic parameters for automation of all steps of the MOB process, we next employed commercially-available kit reagents and protocols with the MOB process to identify the optimal reagents for the individual processing steps. In total, we selected and tested five popular DNA extraction kits, including: NeoGeneStar, Qiagen, MagMax, MagaZorb, and Apostle. Direct comparison was made feasible as all of the selected kits are based upon the same underlying binding chemistry, specifically isolation of DNA from plasma/serum samples via chemically-facilitated adsorption to silica-coated magnetic particles. Furthermore, we used genomic DNA (gDNA) diluted in water as a model sample for our initial comparison to control for sample-specific differences.

All manufacturers’ protocols were amended with two modifications for implementation in the automated platform. First, to prevent evaporation associated with the extended heat incubation processes, mineral oil was added to the surface which obviated the need for manually capping the tubes. We found this solution to be better suited for our application than commonly-used pierceable films, which would need to be applied manually, requiring opening of the enclosure mid-run. With this protocol modification, we were able to perform all the necessary heating steps, including those required for bisulfite conversion, a near-boiling point temperature (98°C) and an extended incubation period (1 hour at 55 °C), without manual input or any observable reagent evaporation. It is worth noting that use of mineral oil benefits from the use of an LHP with the ability to select the pipetting height to enable the aqueous layer to be pipetted below the oil phase.

Most silica-based extraction and purification protocols require careful and complete removal of processing buffers to avoid carryover of downstream assay inhibitors. In fact, our initial results indicated that the automated protocols for several of the extraction kits yielded DNA containing significant levels of PCR inhibitors that impeded downstream analyses and qPCR quantification. These issues were likely the result of inefficient washing due to greater volumes of residual liquid left in the wells during the automated wash steps in comparison to the manual format. To address this issue, we added an additional 80% ethanol wash step to further dilute potential inhibitors before the final elution step (Supplementary Table 2). Since ethanol can also inhibit PCR, we added an evaporation step after the last wash to ensure its complete removal before DNA elution. Overall, addition of this simple step resulted in improved PCR amplification for all the tested kits.

**Figure 3.**
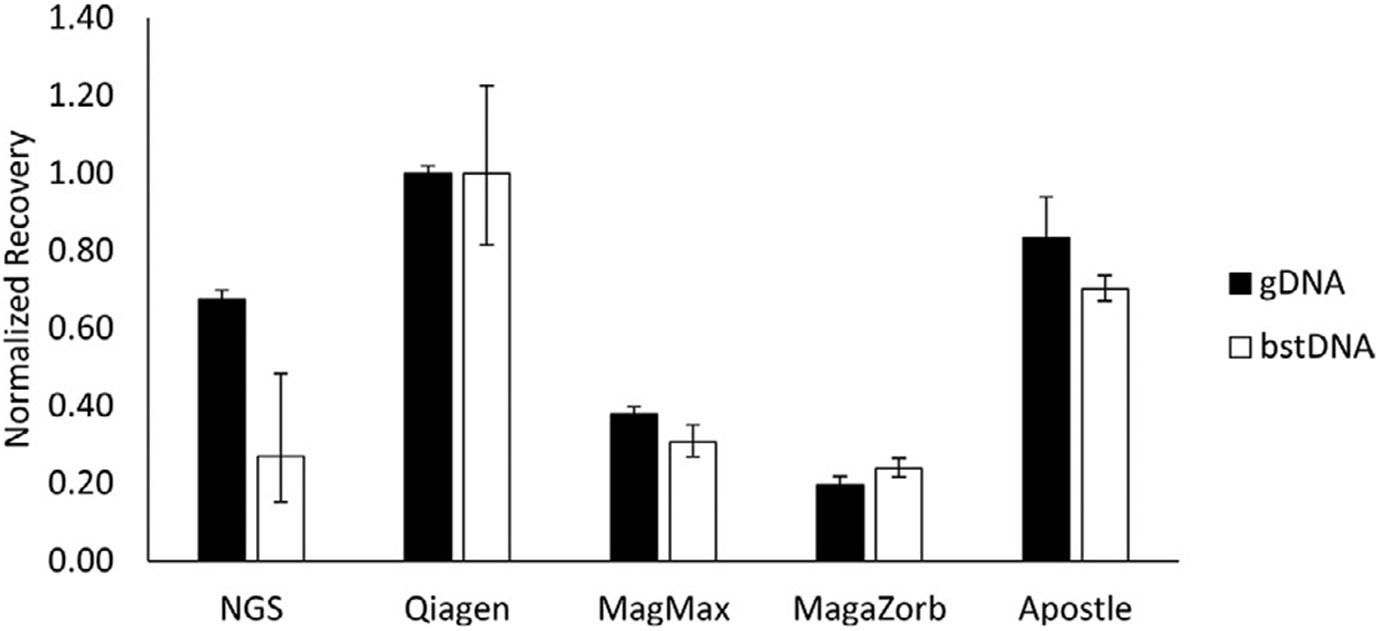
Comparison of normalized DNA yields of automated commercial extraction kits obtained from 200 ng of control gDNA diluted in water. Plot shows normalized DNA yields from automated protocols based on the NeoGeneStar, Qiagen, MagMax, MagaZorb and Apostle kits. The bstDNA yields represent the DNA yield after converting each respective extracted gDNA sample via Zymo Lightning Conversion kit.

After incorporating these amendments, we used standards containing 200 ng of human gDNA suspended in water to compare the relative yield of DNA extraction for each of the respective automated kit protocols. Overall, the Qiagen kit outperformed other kits in terms of both gDNA extraction and bstDNA yields, with a 30% greater yield than the next-best performing kit, which was the Apostle kit (Figure 3). The NeoGeneStar kit performed acceptably in extracting gDNA but did not perform as well for bisulfite conversion. MagMax and MagaZorb kits exhibited the lowest yields in our tests.

**Figure 4.**
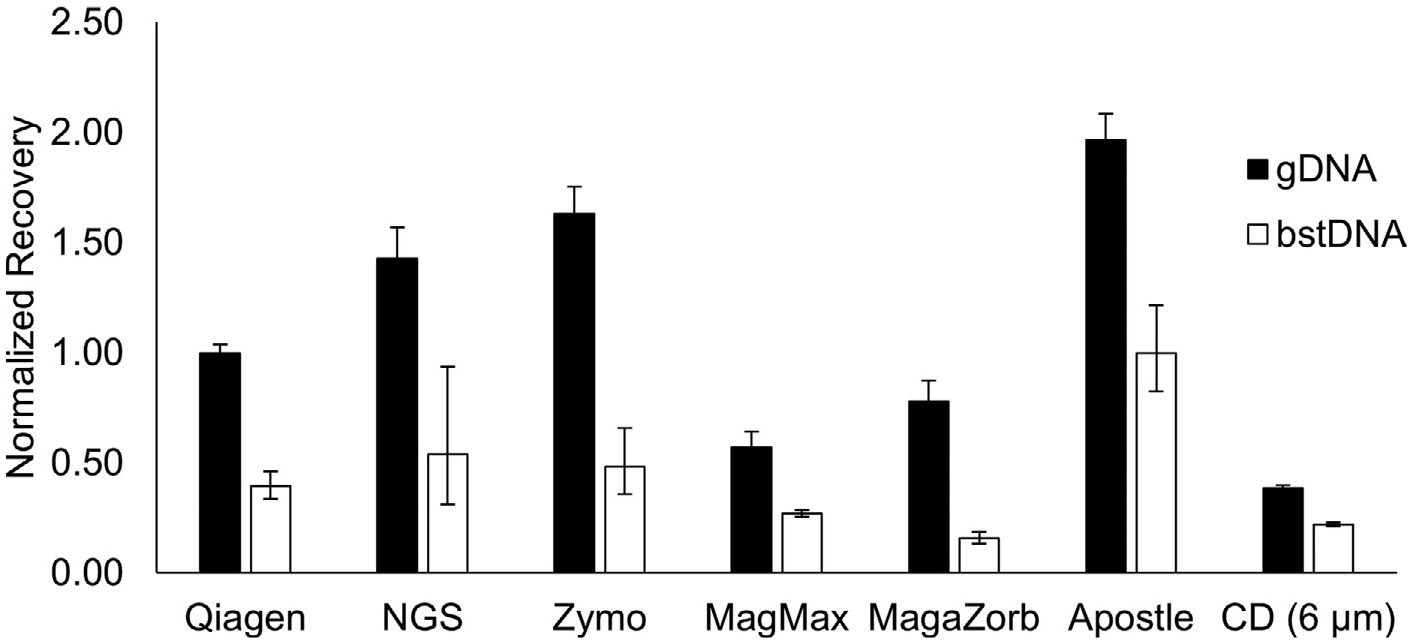
Comparison of DNA yields using silica-magnetic particles from different manufacturer. Extracted DNA yields when using different silica-coated magnetic particles with Qiagen extraction reagents (gDNA) and bisulfite conversion with the Zymo Lightning Conversion kit (bstDNA).

While each of the kits operate under the same basic principle, i.e., silica-based DNA extraction in the presence of chaotropic salts, our comparison indicates that the efficiency of the process can vary widely depending on the buffer formulations and particles employed in each respective kit. Consequently, we next sought to examine the influence of particle composition on overall extraction efficiency. To do this, we utilized the top-performing Qiagen extraction kit reagents but substituted in the magnetic particles from the other commercial kits. We also included particles from two other vendors: Zymo MagBinding particles, as well as notably larger, 6 μm silica-coated magnetic particles from Creative Diagnostics. Once again, we used a fixed 200 ng gDNA sample diluted in water as a standard to compare all kits for DNA recovery. Overall, we found that a number of third-party particles, including those from Zymo, NeoGeneStar and Apostle, were able to improve yields when employed with the Qiagen reagents (Figure 4). Most notably, the Apostle particles outperformed all others, achieving almost 2-fold higher recovery yields than the particles supplied in the Qiagen kit. In contrast, the MagMax, MagaZorb, and Creative Diagnostics particles led to lower yields when implemented with Qiagen reagents. Based on the performance of the reagents in both experiment sets, we determined that the hybrid protocol employing Qiagen buffers with Apostle magnetic particles was able to achieve consistently higher DNA yields than the other tested kit implementations. We also found the performance of NGS and Zymo beads to be acceptable replacements for the Apostle beads with comparable performance. Ultimately, we selected the Zymo MagBinding beads as a practical replacement since these are available for bulk purchase directly from the manufacturer, unlike the Apostle beads which are only bundled with the extraction kit.

**Figure 5.**
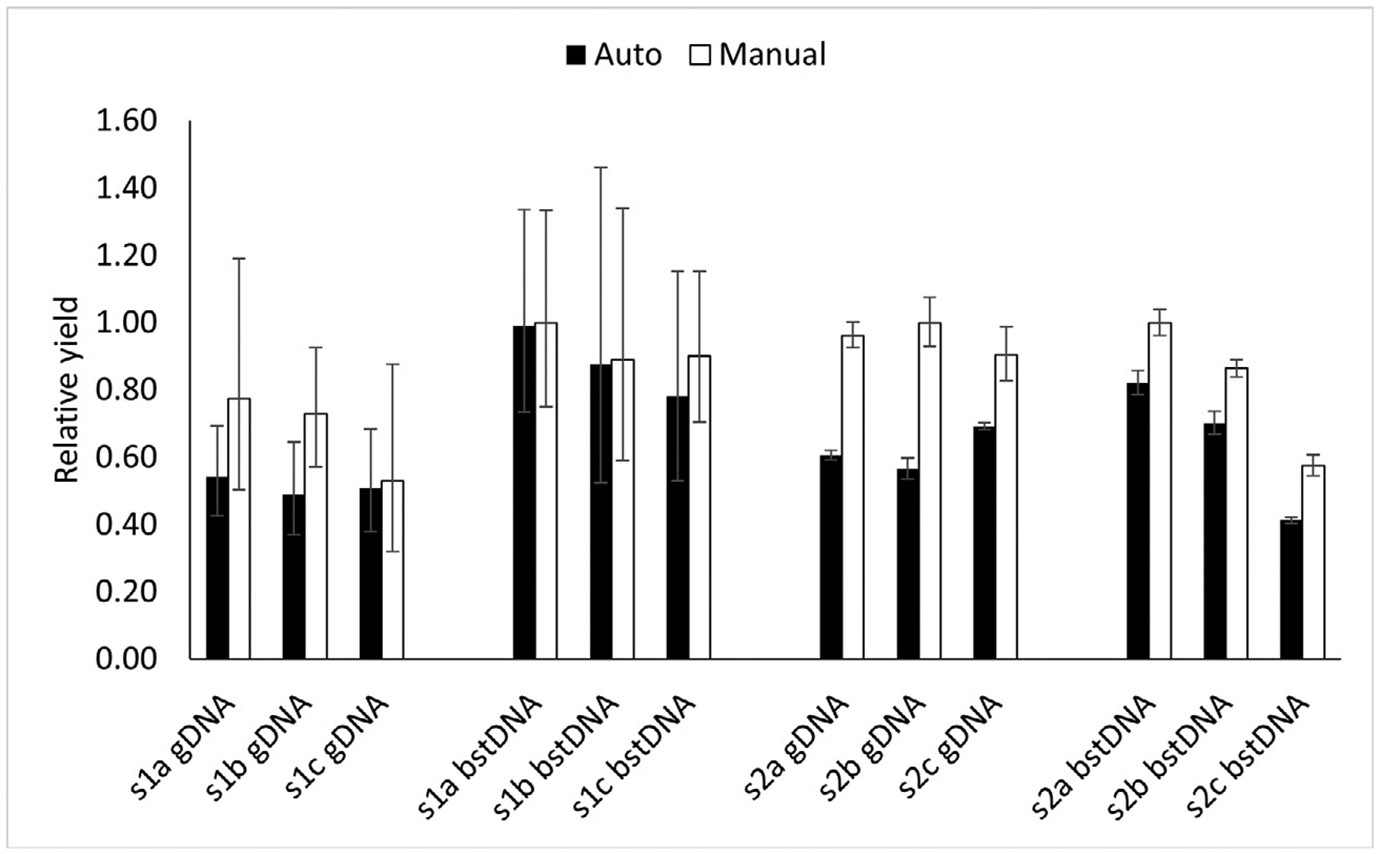
Comparison in DNA yield between manual and automated versions of optimized protocol. Extracted DNA yields from 150 μL of human serum compared after gDNA extraction and after bisulfite conversion (bstDNA).

We next set out to compare the performance of the “optimized protocol” in the automated format to an analogous, manual protocol using the same reagents and particles. For this comparison, we employed pooled human sera to assess performance in a more clinically-relevant sample type, while minimizing subject-to-subject variability in DNA quantity and size. Overall, our results indicated that yields from the automated version of the optimized protocol were comparable to those obtained from manual processing. In particular, the automated process resulted in an average cycle of quantitation (Cq) of 23.66 and a standard deviation of 0.329 while the manual process resulted in an average Cq of 23.19 and a standard deviation of 0.536 across 6 identical samples that were independently processed (Figure 5). Since the Cq means for both cases were seemingly close, we calculated the probability associated with a two-tailed Student’s T-test to determine whether the two populations were statistically different. Surprisingly, not only did the manual process achieve higher yields overall but the p-value from the Student’s T-test (p=0.004) demonstrated this to be statistically significant. We analyzed the 6 samples in 2 datasets (s1a-s1c and s2a-s2c) based on the date they were run and found the p values for s1 (p=0.0678) barely above the commonly accepted threshold to reject statistical difference and s2 (p=0.00005) to be statistically different. We evaluated the F score for both populations to verify equal variance and found for s1 F= 1.056 was below Fcritical=2.818 and for s2 F=1.815 was also below Fcritical=5.050, which validated equal variances for both datasets. Despite this difference, we reasoned that the average difference (0.47 cycles or 39%) is small enough that it may be compensated by a 39% increase in sample size. While we understand for some studies with limited sample volumes it may be an issue, we expect most studies to be able to account for this change in sample volume.

We observed that the machine was not able to remove liquids from the tube as effectively as an experienced user performing the same process manually, typically resulting in a carryover of 5-10 μL between each magnetic decantation step. Likewise, the carryover liquid can ultimately lead to lower final DNA purity and higher concentrations of inhibitors. To overcome this limitation, we included an additional ethanol wash step to effectively mitigate this issue.

Bisulfite-treated DNA yields were also comparable between the manual and automated processes, demonstrating an even smaller difference in recovery. In particular, the automated process resulted in an average Cq of 26.05 with a standard deviation of 0.466, while the manual process resulted in an average Cq of 25.7 with a standard deviation of 0.371. Despite the manual process demonstrating somewhat higher bstDNA yields compared to the automated process (Figure 5), this result was not statistically significant (p = 0.177). When analyzing sample datasets s1 and s2 independently we found s1 (p = 0.645) was not statistically significant but s2 (p = 0.0012) was statistically significant. We evaluated the F score for both populations to verify equal variance and found for s1 F = 1.681 was below Fcritical = 2.818 and for s2 F = 1.576 was also below Fcritical = 5.050, which validated equal variances for both datasets. The difference in mean Cq of 0.22 represents a loss of 21% which can be overcome similarly to gDNA by increasing sample size. The increase in sample size required to obtain similar results in the automated platform is 68% or less and may be further reduced with future refinements. The improvement in relative bstDNA yields can likely be accounted for by more dominant effects associated with bisulfite conversion, such as DNA degradation and loss, that ultimately diminish the observed differences in yields between the manual and the automated process. Overall, our results indicate that the automated protocol typically resulted in yields comparable to those achieved by manual processing, while at the same time maintaining a number of key advantages, including increased throughput and reproducibility independent of user skill, as well as a reduced risk of contamination during the process.

The LHP we selected allows for flexible programing and modification of this protocol. This allows the relevant portions of the current protocol to be used with other methylation assays such as bisulfite-free enzymatic conversion[37,38] and sequencing library preparation with minimal programming changes. The platform can also be incorporated into assays such as digital droplet PCR (ddPCR) as long as the input for such an assay is stored in a 96-well plate, for example, the Bio-Rad QX200 ddPCR system. In general, any assay compatible with silica DNA extraction in a traditional benchtop setting can be incorporated into the automated platform.

There were a number of limitations to our study that warrant discussion. First, we restricted our comparison to a small selection of commercially available extraction kits and magnetic particles. Furthermore, only a limited amount of effort was made to reduce overall processing time and thus the protocol could be further optimized by carefully tuning each step to improve throughput and efficiency, so long as DNA recovery and conversion efficiency are ma intained. We also used only standardized gDNA samples in our kit comparison to isolate the influence of protocol parameters and to directly compare commercial kits. Consequently, the final protocol represents the optimal conditions for processing of these particular sample types. Lastly, we did not comprehensively evaluate the quality of final DNA products in terms of purity and integrity, which may be of particular relevance for downstream analyses such as MS-HRM and long-read sequencing.

In summary, this research demonstrated development of an automated protocol that offers the ability to generate high-quality, bisulfite-treated DNA samples in a high-throughput and clean environment with minimal user intervention and comparable yields to analogous processing performed manually. Furthermore, samples processed by the LHP-based protocol are reproducible regardless of user skill, ostensibly improving the precision and statistical power for downstream sample analyses. Overall, the presented automated protocol provides a useful solution for processing large batches of biological samples for downstream analysis of DNA methylation by a wide variety of methods.

## Supplementary Material

Supplementary material

Supplementary material for this article is available on the Journal of Laboratory Automation Web site at http://jla.sagepub.com/supplemental.

Supplementary material associated with this article can be found, in the online version, at doi:10.1016/j.slast.2021.12.002.
