# Supplementary material for: High-throughput sample processing for methylation analysis in an automated, enclosed environment
Source: SLAS Technol. Author manuscript; Available in PMC 2026 Jul 27. (PMC13403162; doi:10.1016/j.slast.2021.12.002)
Supplement: Supplementary material [file NIHMS2185969-supplement-Supplementary_material.pdf]

Supplementary Material

Supplementary Table 1 Optimized pipetting speed for reagents used in automated MOB protocol

	Aspiration (mm/s)	Dispensing (mm/s)	Mixing (mm/s)	Blow-out
Buffer AL	10	10	N/A	No
Proteinase K	15	10	20	No
cRNA	20	20	20	No
Isopropanol	20	10	N/A	Yes
Magnetic Beads	20	20	75	Yes
Sample Lysate (containing digested sample, buffer AL, proteinase K, cRNA, isopropanol, and magnetic beads)	10	10	11	Yes
Buffer AW	20	10	20	No
80% Ethanol	20	15	N/A	Yes
Elution Buffer	20	10	N/A	Yes
Lightning Conversion Buffer	20	10	20	No
M-Wash Buffer	20	15	20	Yes
L-Desulphonation Buffer	20	10	N/A	No
Supernatant during magnetic binding	4	4	N/A	Yes

Supplementary Table 2 Wash buffer volumes and their effect on inhibition of PCR

gDNA extraction	Buffer AL2	80% Ethanol Wash	Cq
Control	400	-	N/A
Added Ethanol Wash test 1	400	400	27.88
Added Ethanol Wash test 2	400	800	27.57
